# Post gastroenteritis gluten intolerance 

**Published:** 2015

**Authors:** Kamran Rostami, Mohammad Rostami-Nejad, David Al Dulaimi

**Affiliations:** 1 *Gastroenterology Unit, Alexander Hospital Redditch, UK*; 2 *Gastroenterology and Liver Diseases Research Center, Shahid Beheshti University of Medical Sciences, Tehran, Iran*

## Introduction

The spectrum of irritable bowel syndrome (IBS) is narrowing and many conditions previously known/attributed to IBS seem to be related to unrecognised primary or acquired intolerances to nutrient components. Infection might act as triggering factor for inflammatory conditions in susceptible individuals. 

Inflammation of absorptive surface leading to various enzymatic defects, changing the microbiota and increasing intestinal permeability may results in symptoms previously known as post gastroenteritis IBS. It is time to recognise the possible pathophysiology of acquired gluten intolerance that has been miss-treated under the mask of IBS. 

## Case Report

A 32 yrs old female was seen in the gastroenterology clinic with a history of chronic diarrhoea following an episode of gastroenteritis in February 2014. The diarrhoea was associated with urgency, severe dyspeptic like abdominal pain and bloating. She had no significant past medical history. The body mass index was 28. Physical examination was unremarkable. Routine blood tests were normal aside from a mildly raised of ALT. Anti-tissue transglutaminase antibodies for coeliac disease were negative. A cirrhosis screen was negative and an ultrasound scan of the abdomen was normal aside from indicating mild fatty infiltration of the liver. 

Total IgE was normal and the Rast test for mixed food/gluten and wheat was negative. 

OGD and duodenal biopsies were normal and colonoscopy was macroscopically unremarkable apart from a small inflammatory polyp. There was no mucosal inflammation. A clinical diagnosis of post infectious irritable bowel syndrome (IBS) was made. She was treated with a lactose and gluten free diet but no drug therapy was initiated. Symptoms resolved within a few weeks. After a few months she started to re-introduce some gluten in her diet but felt no different and remained asymptomatic. She reported mild epigastric pain, only after consuming certain non-gluten containing foods. The improvement of symptoms on a gluten and lactose free diet would be in keeping with a diagnosis of post gastroenteritis gluten intolerance.

## Discussion

Chronic inflammatory conditions, such as inflammatory bowel disease, are associated with a high prevalence of lactose intolerance (1) and clinicians are also aware that patients with gastroenteritis may develop transient lactose intolerance (2).

**Figure 1:**
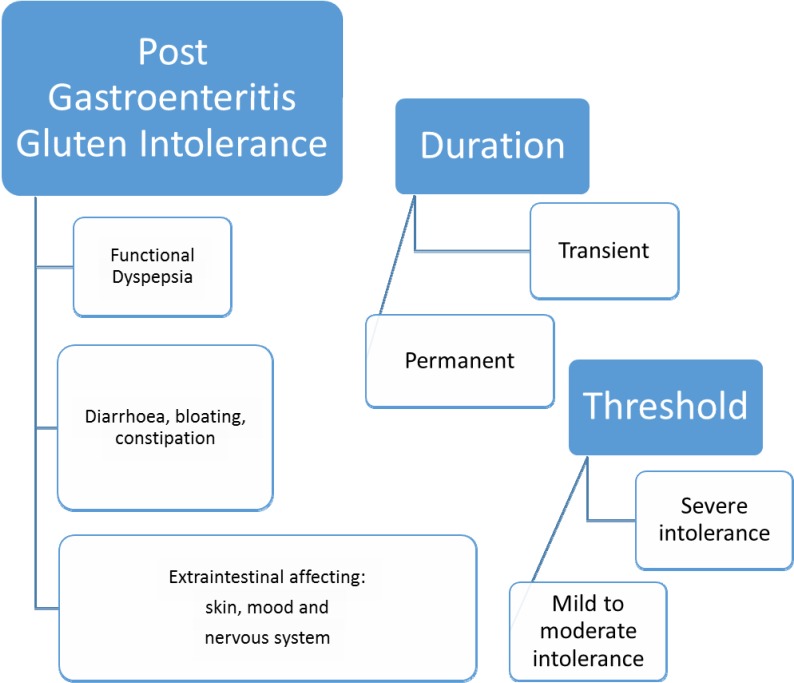
The phenotype of post gastroenteritis gluten intolerance (PGIGI)

**Figure 2:**
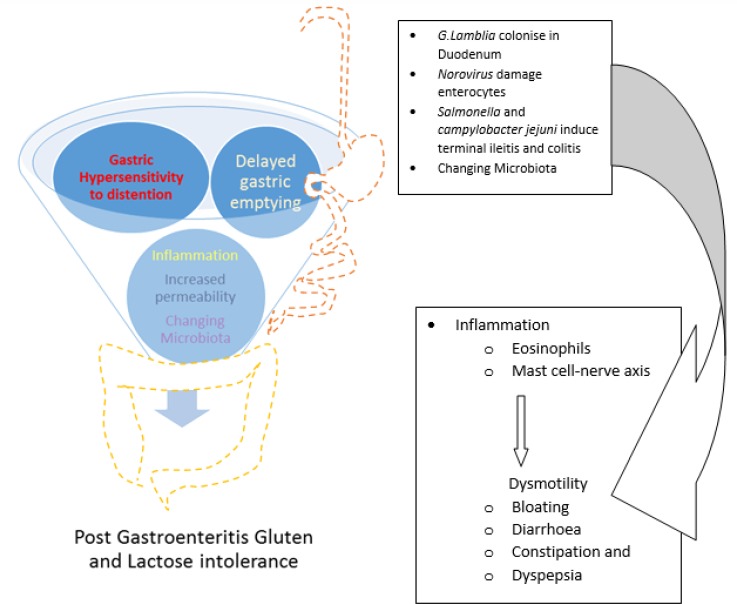
Post gastroenteritis Gluten intolerance: pathophysiology

Viral or bacterial gastroenteritis can cause structural changes to the small bowel mucosa, including locally reduced digestive enzymes activities (3) secondary to the local inflammatory reaction. Peptidase deficiency resulting from infected small bowel can cause accumulation of partially digested gluten peptides and damage the intestinal mucosal cell (4). We speculate that damages and deficiencies might cause transitory or permanent intolerances to gluten and other nutrients (5). This is why some patients might develop intolerance to gluten only over a short period of time and others might be affected permanently. It is possible that some patients develop only a reduced tolerability for gluten and other peptides (See Figure 1). The site of infection in the gut may lead to the type of symptomatology expressed; involvement of the small bowel and colon may cause IBS-like symptoms, whereas involvement of the stomach and duodenum may cause functional dyspepsia (6) (Figure 2).

Transient or permanent post gastroenteritis gluten intolerance might be a common unrecognised clinical condition. Like secondary lactose intolerance, post gastroenteritis gluten intolerance could explain the prolonged symptoms that develop in a group of patients who have suffered from infective gastroenteritis. Patients may present with diarrhoea, bloating, pain, vomiting and dyspepsia. The underlying cause of dyspepsia has been attributed to gastric dysfunctions, like delayed gastric emptying and hypersensitivity to gastric distention (7). It has been reported that gastric emptying and drinking capacity may reduce following *G. lamblia* infection (8) (Figure 2).

Local inflammation in the small bowel may lead to maldigestion of gluten containing carbohydrates and an increase in the amount of undigested carbohydrates in the intestinal lumen. Osmotically active carbohydrates could inhibit water reabsorption in the colon, causing osmotic diarrhoea. In addition undigested gluten containing carbohydrates entering the colon may be digested within the colon by the colonic bacterial flora, leading to fermentation and an increased in colonic gas, causing bloating and excess flatus. This is 

We can only speculate if this patient had a post gastroenteritis gluten intolerance following a GI infection, as part of the spectrum of non-coeliac gluten sensitivity. Non-coeliac gluten sensitivity is an entity separate from coeliac disease with a much higher prevalence (9, 10). The autoantibodies like anti-EMA and/or anti-tTG tests are negative although antigliadin antibodies may be present (11). 

We suggest there may be an important role for the reduction of gluten in the diet as a treatment for these patients, in a manner analogous to the reduction in lactose intake frequently advised by dieticians for symptoms attributed to transient post infectious lactose intolerance.

We suspect that patients who develop lactose and gluten intolerances after an episode of gastroenteritis are labelled as having IBS and can be left untreated for years (12) or given only symptomatic treatment for pain, diarrhoea and constipation rather than advised to reduce their dietary intake of lactose and gluten. By moving toward clear diagnosis and targeted treatment of diseases that are involved in the formation of symptoms, we proportionally are approaching the end of the era of non-specific and unhelpful diagnosis like IBS and post gastroenteritis IBS. Clinician and dietician considering the possibility of a post infectious gastroenteritis irritable bowel syndrome being, in part, due to gluten intolerance may encourage colleagues to consider introducing a trial of a lactose and gluten free diet in suitable candidates after exclusion of celiac disease.
